# Advances in PMP22 sequence variant-associated hereditary neuropathy with liability to pressure palsies: from gene dosage effects to mechanism-informed variant interpretation

**DOI:** 10.3389/fneur.2026.1948887

**Published:** 2026-09-18

**Authors:** Huan Li, Youping Wang, Caixia Zhang, Youwei Liu, Shaoling Xu

**Affiliations:** 1 Department of Neurology, 970th Hospital of PLA Joint Logistic Support Force, Yantai, China; 2 Department of Pathology, 970th Hospital of PLA Joint Logistic Support Force, Yantai, China; 3 Zhaoyuan Maternal and Child Health Service Center (Zhaoyuan Maternal and Child Health Hospital), Yantai, China

**Keywords:** dosage–phenotype continuum, genotype–phenotype correlation, hereditary neuropathy with liability to pressure palsies, HNPP, inherited peripheral neuropathy, PMP22, PMP22 functional impairment, precision medicine

## Abstract

Hereditary neuropathy with liability to pressure palsies (HNPP) is an autosomal dominant neuropathy classically caused by a heterozygous deletion of the 17p11.2-p12 region encompassing PMP22. However, PMP22 sequence variants can produce HNPP-like or overlapping phenotypes, complicating variant interpretation. This narrative review synthesizes clinical, genetic, functional, and experimental evidence relevant to PMP22 sequence variant-associated HNPP. PMP22 sequence variants may cause quantitative loss of functional protein or qualitative impairment through defective folding, trafficking, membrane localization, or MPZ/P0 interaction. We propose a descriptive two-dimensional framework that considers effective PMP22 functional activity and, where experimentally supported, variant-specific proteotoxic or dominant-negative burden; it complements rather than replaces established mechanistic terms such as haploinsufficiency. Clinically, sequence variants should be interpreted in relation to the pressure-palsy phenotype, the distribution of electrophysiological abnormalities, and variant-specific evidence. MLPA remains the first-line test for typical HNPP, whereas PMP22 sequencing and broader panels are relevant in deletion-negative or atypical cases. Current management remains preventive and supportive; molecular approaches remain preclinical. Future progress will require natural-history cohorts and variant-specific cellular and animal models.

## 1 Introduction

Hereditary neuropathy with liability to pressure palsies (HNPP) is an autosomal dominant disorder characterized by recurrent, transient mononeuropathies or multifocal peripheral nerve palsies triggered by minor compression, stretching, or localized mechanical stress. The classic clinical manifestations include weakness, numbness, and paresthesia within the distributions of nerves that are particularly susceptible to entrapment or compression, including the common peroneal, ulnar, median, and radial nerves, as well as the brachial plexus. Symptoms often recur but, in most patients, resolve partially or completely over a period ranging from days to months (1). Epidemiological studies estimate that the prevalence of HNPP is approximately 16 per 100,000 individuals. However, the true prevalence is likely to be substantially underestimated owing to marked phenotypic heterogeneity, mild or asymptomatic presentations in some patients, and frequent misdiagnosis as having acquired entrapment neuropathies, inflammatory demyelinating neuropathies, or other inherited neuropathies. Notably, newborn genetic-screening data indicate that the frequency of 17p11.2 deletion is approximately 1 in 1,700 individuals, corresponding to approximately 58.9 per 100,000 individuals. These findings suggest that the true disease burden of HNPP may be considerably greater than that indicated by clinically diagnosed cases (1, 2).

The classic genetic basis of HNPP is a heterozygous deletion of the 17p11.2-p12 chromosomal region encompassing the PMP22 gene. However, before widespread molecular testing became available, HNPP was also recognized in patients based on characteristic clinical manifestations and electrophysiological findings without genetic confirmation. PMP22 is a key dosage-sensitive gene required to maintain the structural and functional integrity of peripheral nerve myelin. Duplication of PMP22 causes Charcot–Marie–Tooth disease type 1A (CMT1A), whereas a heterozygous deletion leads to HNPP. By contrast, PMP22 sequence variants can give rise to a broad spectrum of phenotypes of varying severity, ranging from HNPP-like presentations and Charcot–Marie–Tooth disease type 1E (CMT1E) to Dejerine-Sottas syndrome (DSS) and congenital hypomyelinating neuropathy (3–5). A PRISMA-based systematic review by Cesaroni et al. (3) that included 127 studies and 4,493 genetically confirmed patients with PMP22-related neuropathies provided a comprehensive overview of this disease spectrum: CMT1A accounted for 75.4% of cases, HNPP for 20.9%, CMT1E for 2.6%, and DSS for 1.2%. In that systematic review, 1.4% of the HNPP cases included in the reviewed literature were attributed to PMP22 sequence variants (3). The phenotypic spectrum of PMP22-related disorders has an important quantitative component: reduced functional PMP22 activity is associated with HNPP-like phenotypes, whereas increased PMP22 dosage causes CMT1A. However, a single linear dosage-phenotype axis is insufficient to account for all sequence variants. In particular, variants causing protein misfolding, endoplasmic-reticulum stress, abnormal protein interactions, dominant-negative effects, or toxic gain of function may produce severe phenotypes that are not predicted by PMP22 abundance alone. We therefore propose a two-dimensional framework in which effective PMP22 functional activity and variant-specific proteotoxicity jointly contribute to disease severity and phenotype.

In this narrative review, PMP22 sequence variants include single-nucleotide substitutions, splice-site variants, deep intronic variants, and small insertions or deletions. A variant was considered HNPP-associated when the reported phenotype included recurrent or episodic focal neuropathies at sites susceptible to compression, with supportive electrophysiological evidence of focal demyelination, prolonged distal latency, or conduction slowing at common entrapment sites. When available, segregation of the variant with affected family members was considered supportive evidence for the association between the variant and the HNPP phenotype. Diffuse background sensory or mixed demyelinating-axonal abnormalities were considered supportive when accompanied by multiple focal entrapment abnormalities. Variants reported exclusively in CMT1E, Dejerine-Sottas syndrome, congenital hypomyelinating neuropathy, or nonspecific polyneuropathy were not classified as HNPP-associated. They are discussed only as mechanistic comparators and are explicitly labeled as such.

## 2 Genetic basis and molecular mechanisms

### 2.1 Dosage sensitivity of the *PMP22* gene and Classic HNPP

The *PMP22* gene is located at chromosome 17p11.2 and encodes a 22-kDa tetraspan membrane glycoprotein. PMP22 is predominantly expressed in compact myelin within the peripheral nervous system, where it accounts for approximately 2–5% of total myelin protein (5). Structurally, PMP22 interacts with myelin protein zero (MPZ/P0) through its transmembrane domains to form a protein complex that is essential for maintaining the integrity of compact myelin architecture (6, 7). The pathogenic mechanism underlying classic HNPP is *PMP22* haploinsufficiency caused by a heterozygous deletion of *PMP22*, which reduces PMP22 expression by approximately 50%. This reduction compromises the structural stability of myelin, disrupts the organization of tight and adherens junctions, and increases myelin permeability, thereby rendering peripheral nerves more susceptible to mechanical stress (8). The heterozygous *Pmp22* knockout mouse model (*Pmp22*+/−), generated by Adlkofer et al. (9), successfully recapitulated the morphological and electrophysiological features of human HNPP, including progressive demyelination and focal myelin swellings, termed tomacula, thereby providing key experimental evidence for the PMP22 dosage-insufficiency hypothesis of HNPP (9). In a standardized nerve-compression experiment, a 60–70% conduction block was induced significantly faster in Pmp22+/− than in Pmp22+/+ mice, and recovery after compression was delayed. Histological analyses showed focal axonal constrictions within tomacula, whereas no segmental demyelination, overt disruption of molecular architecture, or alteration in sodium-channel density was detected under the examined conditions (10). These findings demonstrate reduced resistance of Pmp22-deficient nerves to mechanical compression. The authors proposed that tomacula-associated axonal constriction may increase axial resistance to action-potential propagation and thereby lower the threshold for conduction block; however, this causal link remains a mechanistic interpretation rather than a directly proven mechanism (10).

Importantly, PMP22 haploinsufficiency is not restricted to 17p11.2-p12 deletions. Nonsense, frameshift, canonical splice-site, and pseudoexon-activating variants can reduce output from one PMP22 allele and should be described as conventional haploinsufficiency. In this context, haploinsufficiency refers specifically to reduced gene dosage or expression from one allele, whereas effective functional insufficiency describes the resulting reduction in functional PMP22 activity at the myelin level. We therefore use “effective functional insufficiency” only as a complementary, phenotype-oriented framework, not as a replacement for haploinsufficiency. It refers to insufficient functional PMP22 activity at compact myelin irrespective of whether the mechanism is a quantitative reduction in protein abundance or a qualitative impairment despite preserved abundance. The latter includes missense variants that impair folding, trafficking, membrane incorporation, or interaction with MPZ/P0. The term thus provides value where expression alone is unable to distinguish normal from impaired function, while preserving haploinsufficiency as the precise mechanistic description for variants whose primary effect is loss of expression.

### 2.2 Functional classification and pathogenic mechanisms of PMP22 sequence variants

Unlike those underlying classic deletion-associated HNPP, the pathogenic mechanisms of HNPP associated with sequence variants are highly dependent on variant type and location. Based on previous clinical reports and variant-specific experimental studies investigating PMP22 expression, protein folding, intracellular trafficking, membrane localization, and PMP22–MPZ interaction, representative PMP22 sequence variants can be organized according to their predominant molecular effects and the strength of variant-specific evidence (Table 1). Table 1 summarizes variant-level clinical, genetic, functional, and classification evidence for representative PMP22 sequence variants. It includes variants reported in HNPP as well as selected PMP22-related neuropathies used only as mechanistic comparators; the latter are not interpreted as evidence defining the HNPP phenotype.

**Table 1:** Variant-level clinical, segregation, functional, and classification evidence for representative *PMP22* sequence variants.

PMP22 variant	Phenotype evidence^†^	Genetic evidence^‡^	Variant-specific functional evidence	Current clinical classification^§^	Predominant mechanistic interpretation	Ref.
c.179+1G>C (p.?)	Typical HNPP; meets review HNPP criteria	Familial segregation with affected relatives; rare/absent in controls examined in the original study	Abnormal transcript demonstrated at RNA level with marked reduction of functional PMP22 mRNA	No independent clinical category assigned in this review	Splice-mediated quantitative loss of function/haploinsufficiency	(11)
c.178+2T>C (p.?)	Typical HNPP, including pediatric presentation; meets review HNPP criteria	Affected child and father; family size does not permit estimation of penetrance; population data limited	Aberrant splicing with loss of normal functional output	No independent clinical category assigned in this review	Splice-mediated quantitative loss of function/haploinsufficiency	(12)
c.370delT (p.Trp124Glyfs*31)	Atypical HNPP with painful lumbosacral plexopathy; partial/atypical HNPP support	Familial segregation; absent from major population databases available to the original investigators	Frameshift predicts premature termination; no direct variant-specific cellular assay	Reported as pathogenic in the original study using ACMG-based criteria	Predicted quantitative loss of function	(13)
c.227del (p.Ser76fs)^*^	HNPP-associated neuropathy without documented recurrent palsies; partial/atypical HNPP support	Familial occurrence; very rare, with limited population data	Frameshift predicts truncation; direct variant-specific functional confirmation lacking	ClinVar assertions are not uniform across condition-specific records; not independently reclassified here	Predicted loss of function	(37)
c.289del (p.Tyr97fs)^*^	Painful HNPP-associated neuropathy without reported recurrent palsies; partial/atypical HNPP support	Familial occurrence; population data limited	Frameshift predicts truncation; direct variant-specific functional confirmation lacking	Not independently reclassified in this review	Predicted quantitative loss of function	(38)
c.47T>C (p.Leu16Pro)	CMT1E/DSS-spectrum neuropathy; mechanistic comparator, not established HNPP	Disease-associated familial/*de novo* observations; rare	Experimental evidence supports abnormal folding and intracellular/ER retention	Pathogenic in ClinVar; this does not establish an HNPP phenotype	Reduced functional activity with experimentally supported misfolding/proteotoxicity	(16)
c.199G>A (p.Ala67Thr)	HNPP; meets review HNPP criteria	Reported in HNPP-associated cases/families; rare	Preserved plasma-membrane localization with selective impairment of PMP22–MPZ interaction	Likely pathogenic in a ClinVar condition-specific record	Qualitative loss of functional activity due to impaired PMP22–MPZ interaction; proteotoxicity not inferred	(6)
c.353C>T (p.Thr118Met)	Painful peripheral neuropathy/variable CMT-like findings; characteristic HNPP phenotype not established	Variable genotype–phenotype relationships; present in population databases at a frequency higher than expected for a fully penetrant rare dominant allele	Reduced surface trafficking without prominent toxic intracellular aggregate formation	Conflicting ClinVar classifications for HNPP and related neuropathy records	Trafficking defect demonstrated; clinical pathogenicity and HNPP association remain uncertain	(17–19)
c.179-2809A>G (p.?)	Heterozygous HNPP; homozygous DSS. Heterozygous phenotype meets HNPP criteria; homozygous DSS is a comparator	Seven heterozygous individuals with HNPP and two homozygous siblings with DSS in two Avar families; absent from gnomAD v3.1.2 in the original study	Patient RNA and minigene analyses demonstrated 97-bp pseudoexon activation and premature termination	Likely pathogenic in the original study after segregation and RNA-level functional evidence	Splice-mediated loss of function; variant-specific dosage–phenotype relationship, not a general PMP22 dosage rule	(14)

^†^Phenotype evidence combines the reported phenotype with whether it meets the HNPP criteria defined in the Introduction. “Partial/atypical” indicates incomplete documentation of characteristic pressure palsies; CMT1E/DSS-only phenotypes are included solely as mechanistic comparators. ^‡^Genetic evidence summarizes segregation and population data. Absence from a population database is not independently pathogenic; population context was interpreted with reference to gnomAD (39). ^§^Clinical pathogenicity classification is distinct from mechanistic interpretation. ClinVar assertions may differ by condition, submitter, and review level; ACMG/AMP standards and ClinVar are referenced in (40, 41). ^*^Variants use RefSeq NM_000304.4/NP_000295.1 where applicable; p.? denotes no experimentally established protein consequence. Only direct variant-specific experiments are treated as functional evidence. Trafficking or interaction defects are interpreted as reduced functional activity unless independent evidence demonstrates toxicity or dominant-negative behavior. Abbreviations: ACMG/AMP, American College of Medical Genetics and Genomics/Association for Molecular Pathology; CMT1E, Charcot–Marie–Tooth disease type 1E; DSS, Dejerine–Sottas syndrome; HNPP, hereditary neuropathy with liability to pressure palsies; MPZ, myelin protein zero; PMP22, peripheral myelin protein 22.

The mechanistic categories summarized above should not be considered interchangeable. Evidence from PMP22 deletion or reduced-expression models is most directly applicable to deletion-associated disease and to sequence variants that produce a predominantly quantitative loss of PMP22 function. Truncating and splice-altering variants may also reduce functional PMP22 output, although their molecular consequences should be interpreted variant by variant. Missense loss-of-function variants may impair folding, trafficking, membrane localization, or specific protein interactions despite preserved total protein abundance. In contrast, missense variants associated with intracellular accumulation, dominant-negative effects, or proteotoxicity may not be amenable to strategies designed to increase PMP22 expression or restore dosage alone.

#### 2.2.1 Truncating and splice-site variants: classic dosage insufficiency

Nonsense, frameshift, and splice-site variants reduce functional PMP22 protein levels either through nonsense-mediated mRNA decay (NMD) or by producing truncated, nonfunctional proteins. Their pathogenic mechanisms are therefore most closely analogous to those of heterozygous *PMP22* deletions, as both ultimately result in insufficient amounts of functional PMP22 protein. Bellone et al. (11) reported an HNPP family carrying the c.179+1G>C splice-site variant, in which functional *PMP22* mRNA levels were markedly reduced. Fusco et al. (12) described another family harboring the c.178+2T>C splice-site variant, in which a 4-year-old girl and her father both developed clinical manifestations consistent with HNPP. Both identified carriers were clinically affected; however, the small family size does not permit estimation of penetrance (12). Benquey et al. (13) reported a family with the c.370delT frameshift variant, in which the proband presented primarily with painful lumbosacral plexopathy and was initially misdiagnosed owing to coexisting diabetes mellitus.

#### 2.2.2 Deep intronic variants and pseudoexon activation

The variants described above are located within exons or at canonical splice sites. However, a 2026 study by Chausova et al. (14) expanded the mutational spectrum of HNPP associated with sequence variants to include deep intronic variants. In two Avar families from Dagestan, the authors identified a deep intronic variant in intron 3 of PMP22 (c.179-2809A>G) with a SpliceAI score of 0.74. This variant creates a cryptic splice-donor site, leading to aberrant inclusion of a 97-bp pseudoexon derived from intron 3 in the mature transcript and the introduction of a premature termination codon. Among nine carriers, seven heterozygous individuals presented with typical HNPP, whereas two homozygous individuals, male siblings from the same family, developed severe Dejerine-Sottas syndrome (DSS). This pattern was consistent with a recessive presentation of DSS, with both parents being heterozygous and affected by HNPP (14).

The study provides variant-specific RNA-level evidence that the deep-intronic c.179-2809A>G variant activates a cryptic pseudoexon and disrupts normal PMP22 splicing. Because such variants are not detected by conventional exon-focused sequencing, analysis of noncoding PMP22 regions may be considered in selected patients with a convincing HNPP-like phenotype who remain genetically unresolved after deletion testing and routine sequencing. However, identification of a rare intronic variant alone is insufficient to establish pathogenicity; appropriate segregation analysis and transcript-level functional confirmation should be sought whenever feasible. The reported families are also consistent with a variant-specific functional-dose relationship, as heterozygous carriers presented with HNPP whereas the two homozygous siblings developed DSS. Nevertheless, these observations derive from only two families from the same ethnogeographic population and include only two homozygous individuals, and therefore should not be interpreted as establishing a general dosage–phenotype rule for PMP22 sequence variants. Rather, they illustrate a dosage-related effect for this specific splice-disrupting loss-of-function variant. The activated pseudoexon also provides a defined molecular target for mutation-specific splice modulation. The reported modU7snRNA experiments offer preclinical proof of concept for splice correction, but their implications remain variant-specific and require further validation.

#### 2.2.3 Missense variants: mechanistic diversity

The effects of missense variants depend substantially on their topological position within the protein and the biochemical nature of the amino acid substitution. Their pathogenic mechanisms can be broadly classified into the following categories. First, missense variants may cause protein misfolding and endoplasmic-reticulum retention. Some missense variants disrupt proper PMP22 folding, causing the mutant protein to be recognized by the endoplasmic reticulum (ER) quality-control system and retained in the ER. The misfolded protein is ultimately degraded via the ubiquitin–proteasome pathway, thereby indirectly reducing functional PMP22 protein levels. D’Urso et al. (15) described abnormal accumulation of mutant PMP22 within the ER-Golgi intermediate compartment, whereas Sakakura et al. (16), using structural biology approaches, demonstrated the disruptive effect of the Leu16Pro variant on PMP22 folding. Second, abnormal membrane insertion and intracellular trafficking may contribute to pathogenesis. Certain missense variants within transmembrane domains can interfere with normal membrane insertion and intracellular trafficking, preventing their correct localization to the plasma membrane and compact myelin, the site of physiological PMP22 function. Third, disruption of protein–protein interactions at the myelin membrane represents an additional mechanism. Pashkova et al. (6) showed that PMP22:c.199G>A (p.Ala67Thr) reaches the plasma membrane but selectively impairs PMP22-MPZ association without disrupting the other tested protein interactions. This finding illustrates that preserved PMP22 abundance and plasma-membrane localization may not restore normal functional activity at compact myelin when a critical myelin-protein interaction is disrupted. Such disruption of a specific protein interaction should be considered a qualitative loss of functional PMP22 activity and, in the absence of direct evidence of cellular toxicity or dominant-negative behavior, should not by itself be classified as proteotoxicity. By contrast, when a variant primarily reduces PMP22 expression, haploinsufficiency remains the more precise mechanistic term.

#### 2.2.4 The T118M variant: a controversial susceptibility allele

PMP22:c.353C>T (p.Thr118Met; T118M) is a variant of uncertain clinical significance. Stefanski et al. (17) showed in cellular and biophysical experiments that T118M PMP22 has reduced surface trafficking without prominent toxic intracellular aggregate formation. These findings provide a potential cellular mechanism but do not establish an HNPP phenotype. Record et al. (18) subsequently emphasized that the available population and clinical data do not establish T118M as a proven cause of neuropathy. Ho et al. (19) reported three individuals with painful peripheral neuropathy and variable CMT-like findings; however, recurrent pressure palsies and a characteristic HNPP electrophysiological pattern were not demonstrated, and two individuals had only minimal electrophysiological abnormalities. This case series is therefore discussed only as evidence that T118M has been reported in peripheral neuropathy, rather than as evidence that it causes HNPP. Jerath et al. (20) described one patient carrying both T118M and the 17p11.2 deletion, but this single observation does not establish a synergistic pathogenic effect. Overall, T118M should not be classified as an HNPP-associated variant.

#### 2.2.5 Mutational spectrum and genotype–phenotype correlations of PMP22 sequence variants

Yano et al. (21) analyzed 3,352 Japanese patients with suspected inherited peripheral neuropathies and identified 30 individuals carrying 24 distinct PMP22 sequence variants. Within this cohort, only the splice-site variants c.78+3G>T and c.79-2A>G were associated with an HNPP phenotype. The remaining variants were associated with polyneuropathy rather than classical HNPP. Although the overall PMP22 sequence-variant cohort differed from patients with PMP22 duplications in age at onset, ambulatory ability, and cranial nerve involvement, these aggregate comparisons reflect the heterogeneous cohort as a whole and should not be interpreted as clinical characteristics of sequence-variant-associated HNPP.

### 2.3 Animal models and signaling pathway studies

#### 2.3.1 Pmp22 heterozygous deletion model

The heterozygous *Pmp22* deletion mouse model (*Pmp22*+/−) is a valuable experimental model for investigating the pathological mechanisms of HNPP (9). This model not only recapitulates the core pathological features of HNPP, including tomacula formation and progressive demyelination, but also reveals alterations in the molecular architecture of myelin. Using high-resolution confocal imaging of teased peripheral nerve fibers, Moss et al. (22) assessed the distribution of adherens-junction components, Connexin29, and nodal-domain markers, including Nav channels, Caspr at paranodes, and Kv1.2 at juxtaparanodes, in CMT1A and HNPP model mice. In HNPP nerves, Caspr plaques were fragmented and extended abnormally along internodes, indicating impaired paranodal compartmentalization; however, these Caspr abnormalities were broadly comparable in magnitude between the HNPP and CMT1A models. In contrast, nodal widening of Nav channels and several Schmidt-Lanterman-incisure abnormalities were more prominent in CMT1A. These molecular architectural changes may contribute to altered axoglial compartmentalization and impaired saltatory conduction, but their direct causal relationship to the focal electrophysiological abnormalities of HNPP has not yet been established (22).

At the signaling-pathway level, two separate studies examined molecular events associated with myelin-junction disruption in Pmp22 deletion models. Guo et al. (8) reported in 2014 that PMP22 deletion disrupts tight and adherens junctions within myelin and increases myelin-barrier permeability. In a subsequent study, Hu et al. (23) showed that myelin-junction disruption is associated with increased F-actin polymerization and activation of p21-activated kinase 1 (PAK1). Pharmacological PAK1 inhibition restored F-actin levels, prevented myelin-junction disruption, and ameliorated nerve conduction failure (23). Krauter et al. (24) further showed in rodent PMP22 gene-dosage models that reduced PMP22 expression was associated with lower PTEN levels and increased PI3K/Akt/mTOR signaling, whereas PMP22 overexpression showed the opposite pattern. In HNPP models, rapamycin reduced tomacula formation and improved grip strength and compound muscle action potential amplitudes. These findings identify the PTEN-PI3K/Akt/mTOR axis as a dosage-responsive mechanism of myelin dysregulation; its relevance to sequence variant-associated HNPP remains to be established. This concept of “functional demyelination”—in which molecular disruption of myelin architecture precedes overt morphological demyelination—provides a potential window for early intervention in HNPP.

However, these signaling-pathway studies were based on heterozygous Pmp22 deletion models, and direct extrapolation to HNPP associated with sequence variants requires caution. Loss-of-function effects resulting from truncating or splice-site variants may involve a similar PAK1-mediated pathway. In contrast, missense variants that exert dominant-negative or toxic gain-of-function effects, including through ER retention or protein aggregation, may activate additional stress pathways, including the unfolded protein response (UPR). Mutant PMP22 proteins may also exert pathogenic effects through aberrant interactions with other myelin proteins independently of PAK1. Therefore, the establishment of mutation-specific cellular and animal models is needed to better elucidate the pathogenic mechanisms of different PMP22 sequence variants. Because these findings were obtained in Pmp22 deletion or reduced-expression models, they should be interpreted as evidence for quantitative PMP22 deficiency rather than as a mechanism established for all PMP22 sequence variants.

#### 2.3.2 Pmp22 super-enhancer regulation

Pantera et al. (25) used CRISPR/Cas9 to delete an approximately 40.5-kb super-enhancer region upstream of the Pmp22 gene, thereby generating a Pmp22-SE heterozygous deletion mouse model. Without direct deletion of the Pmp22 coding region, this model successfully recapitulated key pathological features of HNPP via transcriptional downregulation of Pmp22, including tomacula formation, abnormal F-actin polymerization, axonal loss, and compression-induced conduction block. This study provided the first *in vivo* evidence that the upstream Pmp22 super-enhancer is a critical regulatory element governing Pmp22 expression (25). This model not only offers a more refined tool for studying the Pmp22 dosage–effect relationship but also suggests that subtle variants in the super-enhancer region may underlie HNPP-like phenotypes in some patients who test negative by MLPA. These findings raise the hypothesis that regulatory-region variation may merit future investigation in unresolved cases after routine deletion testing and sequencing.

#### 2.3.3 YAP/TAZ-mediated regulation of PMP22 expression

Moore et al. (26) identified a novel transcriptional mechanism that regulates PMP22 protein levels. In Schwann cells, the transcriptional coactivators YAP and TAZ regulate *PMP22* expression through the Hippo signaling pathway. Genetic attenuation of YAP reduces phosphorylated TAZ (p-TAZ) levels, increases TAZ transcriptional activity, and consequently upregulates PMP22 protein levels in peripheral nerves, thereby improving the pathological features observed in HNPP mouse models (26). This finding links the YAP/TAZ pathway—an important transcriptional regulatory axis in developmental neurobiology—to therapeutic strategies for *PMP22*-related disorders. It also identifies a potential therapeutic target for the development of small-molecule therapies aimed at increasing PMP22 levels through transcriptional regulation. The relevance of these regulatory observations to individual missense variants with impaired trafficking, dominant-negative effects, protein accumulation, or toxic gain of function remains unknown.

## 3 Clinical features and diagnosis

### 3.1 Typical and atypical clinical manifestations

A systematic review by Chen et al. (1) including 124 patients with HNPP provided the most comprehensive phenotypic data to date in the Chinese population. Most cases were associated with the canonical PMP22 deletion (77.4%), while a subset carried other PMP22 sequence variants (22.6%). Therefore, these clinical frequencies should be interpreted as representing the overall HNPP spectrum rather than specific features of sequence variant-associated HNPP. Pain and muscle atrophy were reported in 17.7% of patients, whereas pes cavus was observed in 12.1% (1). These data highlight the broad clinical spectrum of HNPP, which extends well beyond its traditional clinical description.

The phenotypic heterogeneity among patients with a sequence variant-associated HNPP phenotype warrants particular attention. The reported clinical manifestations, genetic characteristics, and available functional evidence of PMP22 sequence variant-associated HNPP are summarized in Table 2. Reported PMP22 sequence-variant cases show heterogeneous phenotypes, including some atypical presentations. In a molecular genetic analysis of 24 Chinese HNPP families, Cao et al. (27) identified pathogenic PMP22 variants by sequencing in patients who were negative for PMP22 deletions. The cohort mainly consisted of classical PMP22 deletion-associated HNPP cases, with one family carrying a pathogenic PMP22 sequence variant, highlighting that atypical cases should not be excluded from further genetic assessment solely on the basis of negative MLPA results (27).

**Table 2:** Evidence reported in deletion-associated HNPP and individual reports of HNPP-associated PMP22 sequence variants.

Dimension	Deletion-associated HNPP (17p11.2 deletion)	Individual reports of HNPP-associated PMP22 sequence variants
Genetic mechanism	PMP22 haploinsufficiency, with approximately 50% reduction in expression (8)	Reduced PMP22 output (haploinsufficiency) or impaired protein function despite preserved expression
Frequency of the pathogenic mutation	Predominant genetic mechanism of HNPP	Rare; sequence variants accounted for 1.4% of HNPP cases in a systematic review (3)
Typical phenotype	Mainly typical pressure-palsy phenotype	Typical and atypical HNPP-associated manifestations, including painful plexopathy in individual families
Tomacula	Widely distributed in both compression-prone and non-compression-prone sites (9)	Not systematically assessed across PMP22 sequence variants.
Electrophysiological features	Diffuse mild-to-moderate demyelination with focal conduction abnormalities (1, 30)	Heterogeneous; focal entrapment-site abnormalities have been reported in individual HNPP-associated splice and truncating variants, but genotype-specific distribution remains uncertain (11–13)
Testing method	MLPA is preferred as the first-line test (1, 3)	Sequencing or inherited neuropathy gene panel testing is required when MLPA is negative (27)
Genetic counseling	Autosomal dominant inheritance with variable clinical expression; electrophysiological abnormalities may be present in minimally symptomatic or clinically unaffected carriers.	Clinical expression may vary across individual sequence variants; robust variant-specific penetrance estimates are unavailable.

The sequence-variant column summarizes illustrative observations from individual reports and small families and is not intended as a pooled comparison with deletion-associated HNPP. The two columns therefore should not be interpreted as representing directly comparable patient populations. Findings reported exclusively in CMT1E/DSS-like neuropathy are not used to define the HNPP phenotype.

In pediatric populations, a study examining PMP22 variants in children with persistent toe walking described a heterogeneous PMP22-related phenotype rather than classical HNPP. Children carrying PMP22 variants may present with persistent toe walking, pes cavus, lumbar lordosis, speech impairment, tremor, and reduced reflexes (28). These findings suggest that persistent toe walking and related developmental gait abnormalities may fall within the broader PMP22-related/CMT1E-DSS spectrum and should not be interpreted as typical manifestations of classical HNPP. However, segregation information for individual PMP22 variants remains limited in pediatric cohorts, which should be considered when interpreting genotype–phenotype relationships.

Regarding pain manifestations, Dukefoss et al. (29) found that, among 19 genetically confirmed patients with HNPP, 42.1% had neuropathic pain characterized primarily by burning, aching, or shooting sensations, most commonly involving both feet. Quantitative sensory testing and the quantitative sudomotor axon reflex test suggested functional small-fiber abnormalities, thereby expanding the traditional view that HNPP predominantly affects large myelinated fibers. However, no skin biopsy was performed in this study, and histopathological small-fiber loss was not demonstrated (29). Clinically, pain complaints in patients with HNPP should be promptly recognized and appropriately evaluated to avoid diagnostic delays resulting from the underrecognition of pain symptoms.

### 3.2 Neurophysiological features

Nerve conduction studies and electromyography are central to the diagnostic evaluation of HNPP. Their diagnostic value lies primarily in the distribution of abnormalities rather than in aggregate prevalence estimates derived from heterogeneous nerves and segments. Evaluation should document focal abnormalities at common entrapment sites, particularly prolonged median distal motor latency at the wrist/carpal tunnel, focal slowing of the ulnar nerve across the elbow, and, where clinically indicated, peroneal nerve involvement at the fibular head. The coexistence of abnormalities at more than one entrapment-prone segment is more informative than a single global measure of reduced conduction velocity.

Beyond focal entrapment abnormalities, HNPP may show a diffuse subclinical background neuropathy, including reduced sensory action potential amplitudes and mixed demyelinating-axonal abnormalities. In a study of patients with 17p11.2 deletion-associated HNPP, age-associated deterioration of motor compound muscle action potentials was observed predominantly in nerves vulnerable to repetitive compression, including the median, ulnar, and peroneal nerves, whereas sensory abnormalities were more pronounced but showed less clear age dependence (30). Thus, the combination of multifocal entrapment-site abnormalities and diffuse background nerve involvement can support the diagnosis of HNPP. Reported electrophysiological findings in PMP22 sequence variant-associated HNPP are heterogeneous and are largely derived from individual families or small case series. Some splice-site and truncating variants have been associated with focal abnormalities at entrapment sites similar to classical HNPP, whereas other sequence variants may present with broader demyelinating or mixed electrophysiological patterns. Therefore, electrophysiological findings in sequence variant-associated cases should not be interpreted independently of clinical phenotype and variant-specific evidence. For sequence variant-associated HNPP, electrophysiological findings should be interpreted together with the clinical pressure-palsy phenotype and variant-specific evidence.

### 3.3 Imaging assessment

Imaging is not routinely required for the diagnosis of HNPP. Nerve ultrasound may demonstrate focal enlargement at entrapment sites and may be considered as an adjunctive test in selected atypical cases (31). Armato et al. (32) performed a systematic review and meta-analysis of radiomics applied to peripheral nerve disorders more broadly. Neither this review nor the available HNPP ultrasound literature specifically validates imaging in PMP22 sequence variant-associated HNPP; imaging findings should not be interpreted as diagnostic of this subgroup.

### 3.4 Diagnostic strategy and differential diagnosis

Based on the available evidence, we propose the following stratified diagnostic approach (see Figure 1).

**Figure 1:**
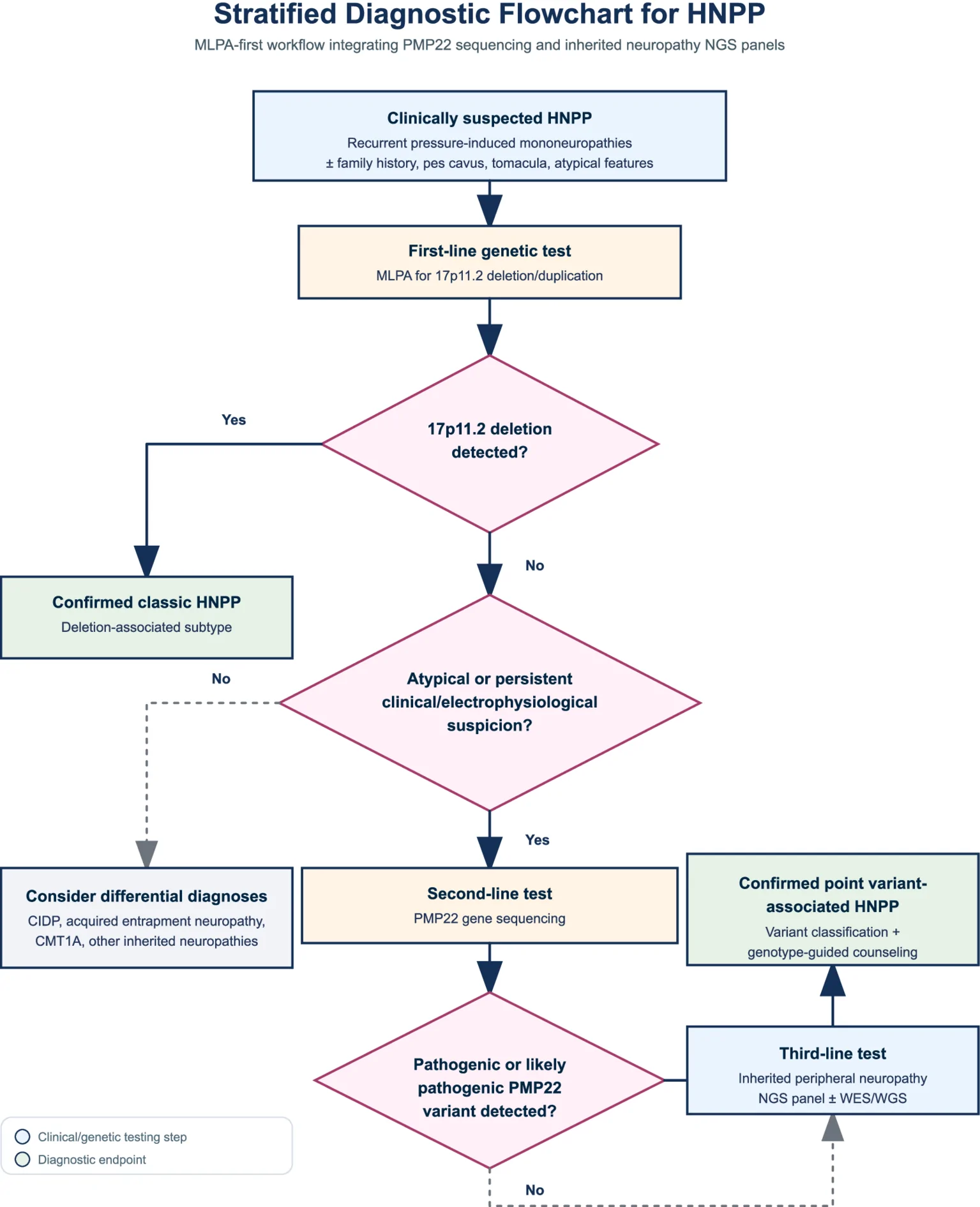
Stratified diagnostic flowchart for HNPP.

MLPA should remain the first-line genetic test in patients with a characteristic HNPP phenotype, particularly recurrent pressure-provoked focal neuropathies with electrophysiological abnormalities at entrapment-prone sites. PMP22 sequencing is appropriate when MLPA is negative but the clinical and electrophysiological phenotype remains compatible with HNPP or an HNPP-overlap phenotype. A broader inherited-neuropathy NGS panel should be considered when PMP22 sequencing is negative, when the phenotype is early-onset, severe, diffuse, or otherwise atypical for HNPP, or when additional systemic or syndromic features are present. In particular, TTR testing should be considered in adults with progressive length-dependent neuropathy accompanied by autonomic symptoms, cardiomyopathy, unexplained weight loss, or bilateral carpal tunnel syndrome. Heterozygous SH3TC2 variants have been reported in a small number of patients with an HNPP-like phenotype; this preliminary observation supports the use of broader panels in selected unresolved cases but does not establish SH3TC2 as a routine targeted test (33).

HNPP should be differentiated from both inherited and acquired disorders with focal or multifocal neuropathies. CMT1A typically produces relatively uniform and symmetric slowing across motor nerves and segments, including non-entrapment segments. In contrast, HNPP more often shows disproportionate slowing, prolonged distal latency, or conduction block at entrapment-prone segments, such as the median nerve at the wrist and the ulnar nerve across the elbow, superimposed on a mild diffuse background neuropathy. Multifocal motor neuropathy should be considered in patients with asymmetric, predominantly motor deficits and conduction block outside typical entrapment sites, particularly when sensory studies are preserved. Focal or multifocal CIDP should be considered when the clinical course is acquired and subacute or progressive and electrodiagnostic evidence of demyelination extends beyond typical compression-prone segments. Assessment should follow the 2021 EAN/PNS CIDP diagnostic criteria, integrating the clinical phenotype, the distribution and pattern of nerve-conduction abnormalities, supportive investigations, and exclusion of inherited neuropathy (34). Cerebrospinal-fluid protein elevation, imaging abnormalities, and treatment response are supportive findings only and should not be used as independent primary discriminators between CIDP and HNPP. This distinction is important because inherited neuropathies with diffuse or multifocal demyelinating abnormalities may be misdiagnosed as CIDP. Transthyretin amyloidosis should be considered when focal neuropathies coexist with a progressive sensorimotor-autonomic neuropathy or relevant systemic manifestations, such as cardiomyopathy, unexplained weight loss, or bilateral carpal tunnel syndrome. TTR genetic testing should be considered when hereditary ATTR amyloidosis is clinically suspected.

## 4 A two-dimensional mechanistic framework for PMP22-related disorders

PMP22-related disorders have traditionally been classified into discrete clinicogenetic entities: CMT1A caused by PMP22 duplication, HNPP caused by PMP22 deletion, CMT1E caused by PMP22 sequence variants associated with severe demyelinating neuropathy, and DSS caused by PMP22 sequence variants associated with severe congenital hypomyelinating neuropathy (3, 5). In this section, CMT1E and Dejerine-Sottas syndrome are considered as mechanistic comparators within the broader PMP22 disease spectrum and are not classified as HNPP in the absence of a characteristic pressure-palsy phenotype. Although these disorders share a quantitative component related to PMP22 function, phenotype severity cannot be represented on a single linear axis from reduced to increased PMP22 activity. We therefore propose a two-dimensional mechanistic framework. The horizontal axis represents effective PMP22 functional activity at compact myelin, ranging from reduced to increased activity. This dimension encompasses not only quantitative changes in PMP22 abundance but also qualitative defects that reduce physiological PMP22 function, such as impaired trafficking, defective membrane incorporation, or disruption of specific interactions with myelin proteins. The vertical axis represents variant-specific cellular toxicity or dominant-negative burden and should be applied only when there is direct experimental evidence of toxic intracellular accumulation, proteotoxic stress, endoplasmic-reticulum stress, aggregation, dominant-negative behavior, or another toxic gain-of-function mechanism. Importantly, impaired trafficking or loss of a specific protein interaction, in the absence of such evidence, should primarily be interpreted as reduced effective PMP22 functional activity rather than as increased proteotoxic burden. Deletion-associated HNPP and sequence variants producing predominantly loss-of-function effects are expected to lie primarily along the functional-activity axis, whereas CMT1A reflects increased PMP22 dosage. Selected missense variants associated with severe CMT1E/DSS-like phenotypes may additionally extend along the vertical axis, but only when variant-specific experimental evidence supports cellular toxicity, proteotoxic stress, toxic gain of function, or dominant-negative behavior. Thus, the two dimensions are mechanistically distinct but may coexist in individual variants when supported by experimental evidence.

Clinically, the continuum model implies that PMP22 sequence variants should be considered in the differential diagnosis of atypical demyelinating neuropathies, even when the electrophysiological pattern does not fully conform to that of classic HNPP. In children presenting with “soft signs,” such as persistent toe walking, pes cavus, and reduced reflexes, the possibility of a broader PMP22-related neuropathy, including CMT1E/DSS-spectrum phenotypes, should be considered rather than classical HNPP alone (28). From a research perspective, the continuum model provides a conceptual framework for functional genomics studies. By systematically comparing PMP22 variants associated with different levels of functional activity, future studies may more precisely define the quantitative relationships among PMP22 dosage, myelin architecture, electrophysiological parameters, and clinical manifestations (see Figure 2).

**Figure 2:**
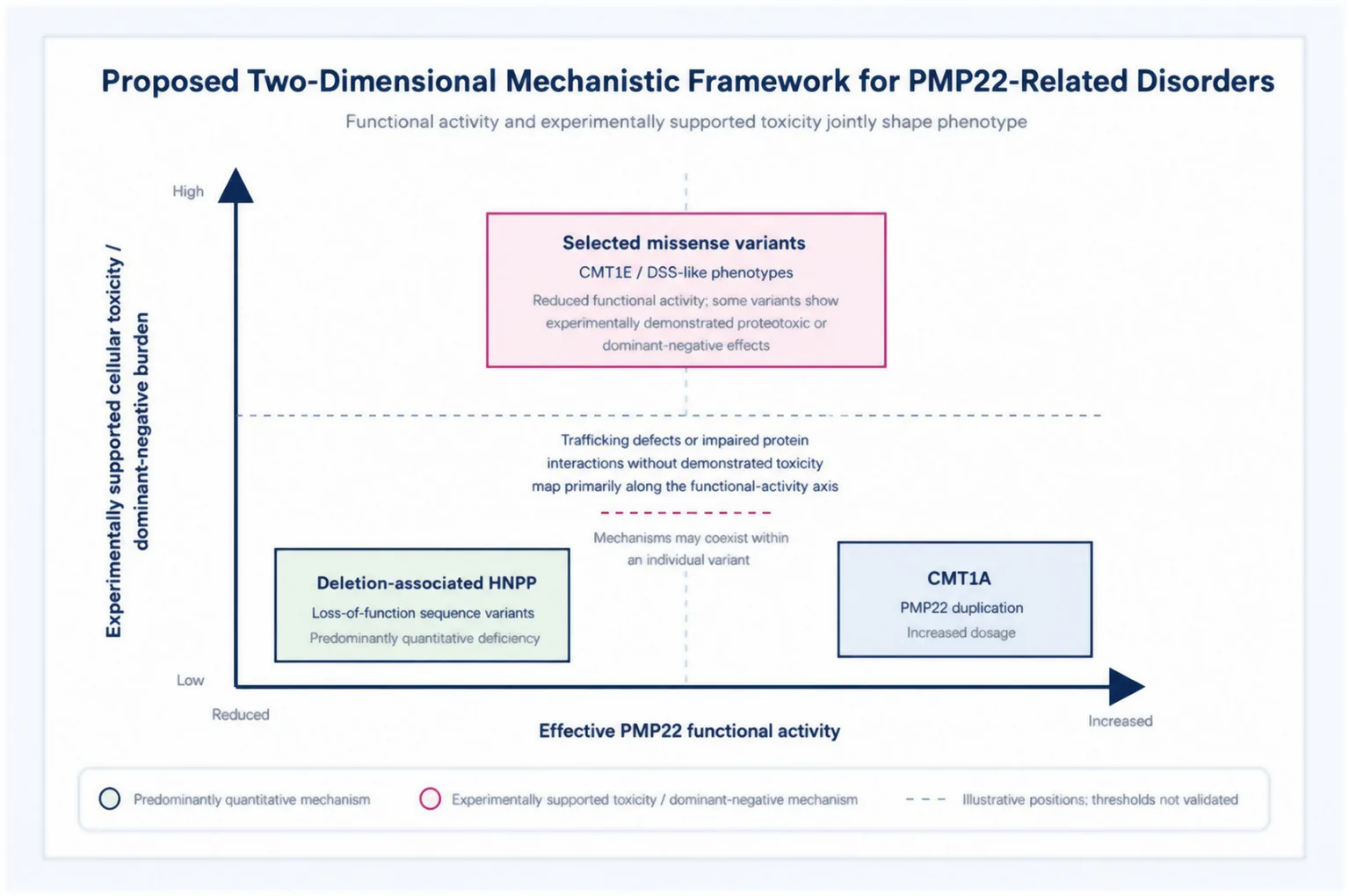
Proposed two-dimensional mechanistic framework for PMP22-related disorders. The horizontal axis represents effective PMP22 functional activity, whereas the vertical axis represents experimentally supported cellular toxicity or dominant-negative burden. Defective trafficking, membrane incorporation, or specific protein interactions primarily indicate reduced functional activity unless independent evidence demonstrates proteotoxicity or dominant-negative behavior. Positions are illustrative and do not imply validated quantitative thresholds.

## 5 Management strategies and therapeutic perspectives

### 5.1 Current management strategies

At present, there is no specific disease-modifying therapy for HNPP, and management focuses primarily on prevention, supportive care, and symptomatic treatment. The core strategy is to avoid precipitating factors, including prolonged kneeling, leaning on the elbows, crossing the legs, and other postures that may compress peripheral nerves. Patients are also advised to wear loose-fitting clothing and footwear and to maintain an appropriate body weight to reduce mechanical stress on peripheral nerves. For patients engaged in occupations involving prolonged kneeling or professional musical performance, occupational counseling and ergonomic modifications are particularly important (1). Management of acute episodes includes rest, physical therapy, and the short-term use of nonsteroidal anti-inflammatory drugs, when necessary. In patients with clinically significant neuropathic pain, medications such as gabapentin or pregabalin may be considered (29). Accordingly, pathway-based therapeutic strategies discussed below should be regarded as preclinical approaches primarily relevant to quantitative PMP22 deficiency, rather than as treatments applicable to all PMP22 sequence variants.

Because classical HNPP is often mild, episodic, or subclinical, preventive management and symptomatic care remain appropriate for most patients. The threshold for exposing patients to an irreversible or systemically delivered molecular therapy should therefore be high. Any future disease-modifying intervention should be considered only for genetically confirmed, severe and progressive phenotypes with substantial cumulative disability, after demonstration that the variant mechanism is amenable to correction and that PMP22 modulation can be safely controlled.

### 5.2 Precision medicine approaches

Kramarz et al. (35) systematically reviewed the current status and challenges of precision-medicine therapies for inherited peripheral neuropathies. Therapeutic development for PMP22-related disorders is particularly challenging because it requires precise dosage control: PMP22 overexpression causes CMT1A, whereas insufficient expression causes HNPP, resulting in an extremely narrow therapeutic window. Chrysostomaki et al. (36) provided a comprehensive summary of emerging therapeutic strategies for inherited polyneuropathies in the era of precision medicine. The major therapeutic approaches encompass nucleic acid-based and small-molecule or pathway-modulating interventions. The level of evidence differs substantially among these approaches: splice correction has been demonstrated in patient-derived fibroblasts, whereas PAK1 and PI3K/Akt/mTOR pathway modulation have been evaluated mainly in Pmp22 deletion-model rodents. Gene replacement, allele-specific silencing, and direct correction remain theoretical or preclinical proposals for PMP22 sequence variants, and no human interventional study has established the safety or efficacy of a disease-modifying therapy for HNPP.

Nucleic-acid-based splice-modulation strategies may offer mutation-specific therapeutic options. A recent study by Chausova et al. (14) provided a preclinical example of modified U7 small nuclear RNA (modU7snRNA)-based splice modulation in HNPP associated with a deep intronic PMP22 variant. For the pseudoexon activated by the deep intronic c.179-2809A>G variant, the authors designed four modified U7 small nuclear RNAs (modU7snRNAs) targeting the pseudoexon splice sites and splicing-enhancer motifs. In patient-derived fibroblasts, these constructs nearly completely restored normal splicing of wild-type PMP22 mRNA, with the modU7snRNA targeting the pseudoexon acceptor splice site showing the strongest effect (14). These results provide preclinical proof of concept for mutation-specific splice correction, but do not support clinical use of splice-modulation therapy in typical HNPP. If validated *in vivo*, this approach may be most relevant to patients with severe disease caused by the homozygous c.179-2809A>G variant, which is associated with Dejerine-Sottas syndrome, rather than to heterozygous carriers with typical HNPP.

Small-molecule and pathway-modulating therapies are emerging as mechanistically informed therapeutic approaches. Inhibition of PAK1 activity can restore myelin-junction integrity and improve nerve conduction (23), supporting PAK1 as a preclinical small-molecule target in Pmp22 deletion-model HNPP; its relevance to PMP22 sequence-variant-associated disease remains unestablished. For sequence variants that cause protein misfolding, chemical chaperones or modulators of endoplasmic-reticulum stress may have therapeutic potential. The PTEN-PI3K/Akt/mTOR axis is another dosage-responsive pathway with preclinical activity in Pmp22 deletion-model HNPP (see Section I.3.1); however, its therapeutic relevance to PMP22 sequence variant-associated HNPP remains unestablished (24). Any systemic mTOR inhibition would also require careful evaluation of safety, therapeutic window, and long-term tolerability.

At present, none of the variants reviewed warrants gene therapy in clinical practice. For typical heterozygous HNPP-associated loss-of-function variants, including canonical splice-site and truncating variants, preventive management remains appropriate because clinical severity is usually limited and increasing PMP22 expression carries a theoretical risk of overcorrection. In the future, tightly controlled augmentation strategies might be explored only in patients with a severe, progressive phenotype and compelling evidence of quantitative PMP22 deficiency. By contrast, variants with a confirmed toxic gain-of-function or dominant-negative mechanism would not be suitable for gene replacement; allele-specific silencing or direct correction may be conceptually preferable, but remains preclinical. Among the variants discussed in this review, homozygous c.179-2809A>G-associated Dejerine-Sottas syndrome currently represents the clearest variant-specific preclinical rationale for splice correction, although evidence remains limited to the reported families and *in vitro* experimental models.

## 6 Research gaps and future directions

Although substantial progress has been made in the study of HNPP associated with PMP22 sequence variants, several critical gaps remain. Systematic genotype–phenotype correlations remain inadequately characterized. Existing evidence is largely derived from individual case reports and small case series, with limited systematic comparisons across different sequence-variant classes with respect to clinical manifestations, electrophysiological patterns, prognosis, and recurrence risk. The controversy surrounding the pathogenicity of T118M (18, 19) highlights the need for reliable high-throughput functional-validation platforms, including standardized assays of PMP22 expression, membrane localization, MPZ interaction, and endoplasmic-reticulum stress.

Models specific to individual sequence variants are lacking. Current animal and mechanistic studies have primarily focused on PMP22 deletion or overexpression (9, 23). Knock-in mouse models carrying specific sequence variants and patient-derived induced pluripotent stem cell (iPSC)-derived Schwann cell models remain largely lacking. Such models are indispensable for distinguishing the pathogenic mechanisms of different sequence variants, including dosage insufficiency, protein toxicity, and disrupted protein interactions, and for testing mutation-specific therapeutic strategies.

Diagnostic pathways require further standardization. Although this review proposes a stratified diagnostic framework, its evidence base requires prospective validation in multicenter studies. For patients who test negative for deletions but present with atypical HNPP, CMT1E-like phenotypes, or pediatric developmental gait abnormalities, there is still no consensus regarding the indications for, testing sequence of, or health-economic evaluation of *PMP22* sequencing and broader inherited-neuropathy gene-panel testing (1).

A translational gap remains between preclinical proof-of-concept studies and clinical application. Multiple preclinical strategies have shown promise, including PAK1 inhibition (23), PI3K/Akt/mTOR pathway modulation (24), and modU7snRNA-based splice modulation (14). However, a clear path to clinical translation remains unestablished. Future studies should prioritize addressing several translational barriers, including the development of sequence variant-specific *in vitro* and *in vivo* efficacy-evaluation systems; the resolution of technical bottlenecks in Schwann cell-targeted drug delivery; the definition of the dosage–effect relationship and safety window for PMP22 expression modulation; and the establishment of biomarker-based systems for therapeutic monitoring.

Natural-history and prognostic data are scarce. Prospective, longitudinal natural-history studies of patients with HNPP associated with sequence variants are currently lacking. Key unanswered questions include annual attack rates across different variant types, the extent of recovery after episodes, the progression of cumulative disability, and the impact on quality of life. Such studies will be essential for the design of clinical-trial endpoints and the evaluation of future interventions.

## 7 Conclusion

HNPP caused by PMP22 sequence variants represents one of the most heterogeneous and challenging subtypes within the spectrum of PMP22-related disorders. It may present with HNPP-like pressure-induced palsies and multifocal electrophysiological abnormalities, while pathological features such as tomacula have not been systematically characterized across sequence variants. This narrative review advances an integrated framework encompassing pathogenic mechanisms, variant-level evidence interpretation, clinical phenotypes, diagnostic evaluation, and therapeutic stratification. The proposed framework may assist variant-level interpretation but does not replace established mechanistic classifications.

Clinically, PMP22 sequence variants encompass a broad phenotypic spectrum, ranging from classic HNPP-like pressure palsies to CMT1E/DSS-like neuropathies; pediatric developmental gait abnormalities should be considered within the broader PMP22-related spectrum rather than as classical HNPP (28). From a diagnostic perspective, this review proposes a stratified pathway integrating MLPA for copy-number analysis, PMP22 sequencing for sequence-level analysis, and next-generation sequencing (NGS) panels for expanded genetic assessment. From a therapeutic perspective, no PMP22 sequence variant currently warrants gene therapy in clinical practice. For severe progressive variants with demonstrated quantitative PMP22 deficiency, tightly controlled augmentation remains a theoretical future strategy rather than a current clinical option.

Future progress in this field will depend on three key transitions: a mechanistic transition from “variant discovery” to “variant interpretation,” a diagnostic transition from a “single testing strategy” to a “stratified testing strategy,” and a therapeutic transition from “symptomatic management” to “mutation-specific precision therapy.” Achieving these transitions will require larger multicenter cohort studies, the establishment of animal and cellular platforms specific to individual sequence variants, and the development of systematic frameworks for genotype–phenotype correlation analysis.
